# Case Identification of Depression in Inpatient Electronic Medical Records: Scoping Review

**DOI:** 10.2196/49781

**Published:** 2024-10-14

**Authors:** Allison Grothman, William J Ma, Kendra G Tickner, Elliot A Martin, Danielle A Southern, Hude Quan

**Affiliations:** 1 Centre for Health Informatics, Cumming School of Medicine, University of Calgary, CWPH Building, 3280 Hospital Drive NW, Calgary, AB, T2N 4Z6, Canada, 1 4032202779, 1 4032109744; 2 Health Research Methods and Analytics, Alberta Health Services, Calgary, AB, Canada; 3 Department of Community Health Sciences, Cumming School of Medicine, University of Calgary, Calgary, AB, Canada

**Keywords:** electronic medical records, EMR phenotyping, depression, algorithms, health services research, precision public health, inpatient, clinical information, phenotyping, data accessibility, scoping review, disparity, development, phenotype, PRISMA-ScR, Preferred Reporting Items for Systematic Reviews and Meta-Analyses extension for Scoping Reviews

## Abstract

**Background:**

Electronic medical records (EMRs) contain large amounts of detailed clinical information. Using medical record review to identify conditions within large quantities of EMRs can be time-consuming and inefficient. EMR-based phenotyping using machine learning and natural language processing algorithms is a continually developing area of study that holds potential for numerous mental health disorders.

**Objective:**

This review evaluates the current state of EMR-based case identification for depression and provides guidance on using current algorithms and constructing new ones.

**Methods:**

A scoping review of EMR-based algorithms for phenotyping depression was completed. This research encompassed studies published from January 2000 to May 2023. The search involved 3 databases: Embase, MEDLINE, and APA PsycInfo. This was carried out using selected keywords that fell into 3 categories: terms connected with EMRs, terms connected to case identification, and terms pertaining to depression. This study adhered to the PRISMA-ScR (Preferred Reporting Items for Systematic Reviews and Meta-Analyses extension for Scoping Reviews) guidelines.

**Results:**

A total of 20 papers were assessed and summarized in the review. Most of these studies were undertaken in the United States, accounting for 75% (15/20). The United Kingdom and Spain followed this, accounting for 15% (3/20) and 10% (2/20) of the studies, respectively. Both data-driven and clinical rule-based methodologies were identified. The development of EMR-based phenotypes and algorithms indicates the data accessibility permitted by each health system, which led to varying performance levels among different algorithms.

**Conclusions:**

Better use of structured and unstructured EMR components through techniques such as machine learning and natural language processing has the potential to improve depression phenotyping. However, more validation must be carried out to have confidence in depression case identification algorithms in general.

## Introduction

### Background

Depression is a significant factor contributing to the global burden of disease. It contributes significantly to the cost of health care services, with depression treatment services costing an average of CAD $550 (US $406.12) per patient in Alberta, Canada, in the 2007/2008 fiscal year [1]. Depression also carries a significantly higher mortality rate [2]. Surveillance of depression in the population is necessary to understand the needs of patients and allocate limited resources where they are most needed. This surveillance will ultimately allow health care professionals to make more targeted decisions when implementing population-level interventions.

Electronic medical records (EMRs) are a digitized collection of patient records documented by medical professionals. They contain various types of patient information, including test results, demographic data, and information about medication orders, recorded in structured data fields and free-text data, such as discharge summaries and nurses’ notes [3-5]. EMRs were designed to aid individual patient care but are increasingly used for other purposes, such as research and gathering data for precision public health efforts, as they are compiled in large data warehouses [6-9]. An area that will be instrumental in applying EMRs to public health is case phenotyping, which is developing case definitions to identify positive cases of a disorder in EMR data.

Accurate case identification in EMRs is an area where more research needs to be conducted. This is especially true for case identification of psychiatric disorders. Previous reviews of phenotyping algorithms for psychiatric disorders only considered primary care databases as their setting [10,11]. However, these are very different from inpatient EMR systems. For one, hospital inpatients are more likely to identify errors and omissions than patients in outpatient care or primary care [12]. EMR data have been used in research for psychiatric patients in various specific inpatient use cases, including assessing patient safety events in psychiatric inpatient units [13]. Research has also shown that hospitals with electronic psychiatric EMRs had lower readmission rates for psychiatric patients compared to hospitals without electronic records. Similarly, hospitals where psychiatric records were accessible to nonpsychiatric physicians had lower 14- and 30-day readmission rates [14]. In 2015, patients with a mental health diagnosis made up over 11% of hospital separations and 25% of hospital days [15]. Accurate case identification for inpatient stays for this at-risk population can help to identify what treatments have been most successful more efficiently than traditional research methods and could work in personalizing care for a more successful treatment plan.

### Objectives

This study aims to provide an overview of existing algorithms for depression case identification in inpatient EMRs. It examines the performance of the algorithms and how they were constructed to provide guidance to those wishing to use an existing algorithm or to construct new ones.

## Methods

### Identifying Relevant Literature

This review followed the methodology outlined in the PRISMA-ScR (Preferred Reporting Items for Systematic Reviews and Meta-Analyses extension for Scoping Reviews) 2018 statement [16]. First, we used the *ICD-9-CM* (*International Classification of Diseases, Ninth Revision, Clinical Modification*) codes for depression provided by Elixhauser et al [17] to identify relevant terms, then developed a Boolean algorithm using these terms, as well as terms related to EMRs and terms related to case identification (Multimedia Appendix 1). Finally, we searched the following 3 databases: Embase (1974 to May 2023), Ovid MEDLINE (1946 to May 2023), and APA PsycInfo (1806 to May 2023) for peer-reviewed papers and exported the results of the search to a reference manager program (Zotero; Corporation for Digital Scholarship and Roy Rosenzweig Center for History and New Media).

### Selecting Studies

Identified papers were screened in 2 stages. First, titles and abstracts were screened by 2 reviewers working independently to determine whether they met our established eligibility criteria. Papers were included if they were retrieved by the Boolean search and presented a case definition, involved depression and EMRs, were published between January 2000 and May 2023, and were written in English. We excluded papers that only used administrative databases, as this study focused on case phenotyping using EMR data. Next, full papers were reviewed for all abstracts that both reviewers identified as eligible. This review was carried out by 2 reviewers working independently. To be included, studies had to use EMRs for phenotyping and use inpatient data, and the case definition developed had to be for depression. The inpatient data source requirement was added because of differences in coding standards between primary care and inpatient settings. Disagreements at either screening stage were resolved by consensus, and if necessary, a third reviewer was consulted. We searched the references of all included papers for additional eligible papers, which we then screened using the same criteria. The search was designed to include all papers that used an algorithm phenotyping for depression with an EMR. The 2 most common methods were natural language processing (NLP) and machine learning, which were included but were not limited to. The search terms used to identify this category were not specific to a type of algorithm or method of case identification, as the purpose was to include a broad range of variations in phenotypic methodology (Multimedia Appendix 1).

### Extracting Data

We adapted an existing data extraction form (Multimedia Appendix 2, Lee et al [18]) to collect the results of our review. Data were extracted by 1 reviewer and then confirmed by a second reviewer. Components we extracted included study characteristics (country, year, and inpatient or outpatient setting), the specific data source and details of the data, and the validation methodology (eg, medical record review), as well as detailed descriptions of the phenotype developed, the methods used, and the purpose for the case definition. We recorded the performance of the developed algorithms as reported in each study. We recorded the elements of EMRs used, whether other databases or diagnostic codes were used, and whether AI techniques (machine learning and NLP) were used as binary variables. Finally, based on this study’s primary objective, we classified each study into 1 of 3 categories (algorithm development, outcome analysis, and comorbidity analysis).

## Results

### Paper Screening

**Figure 1.**
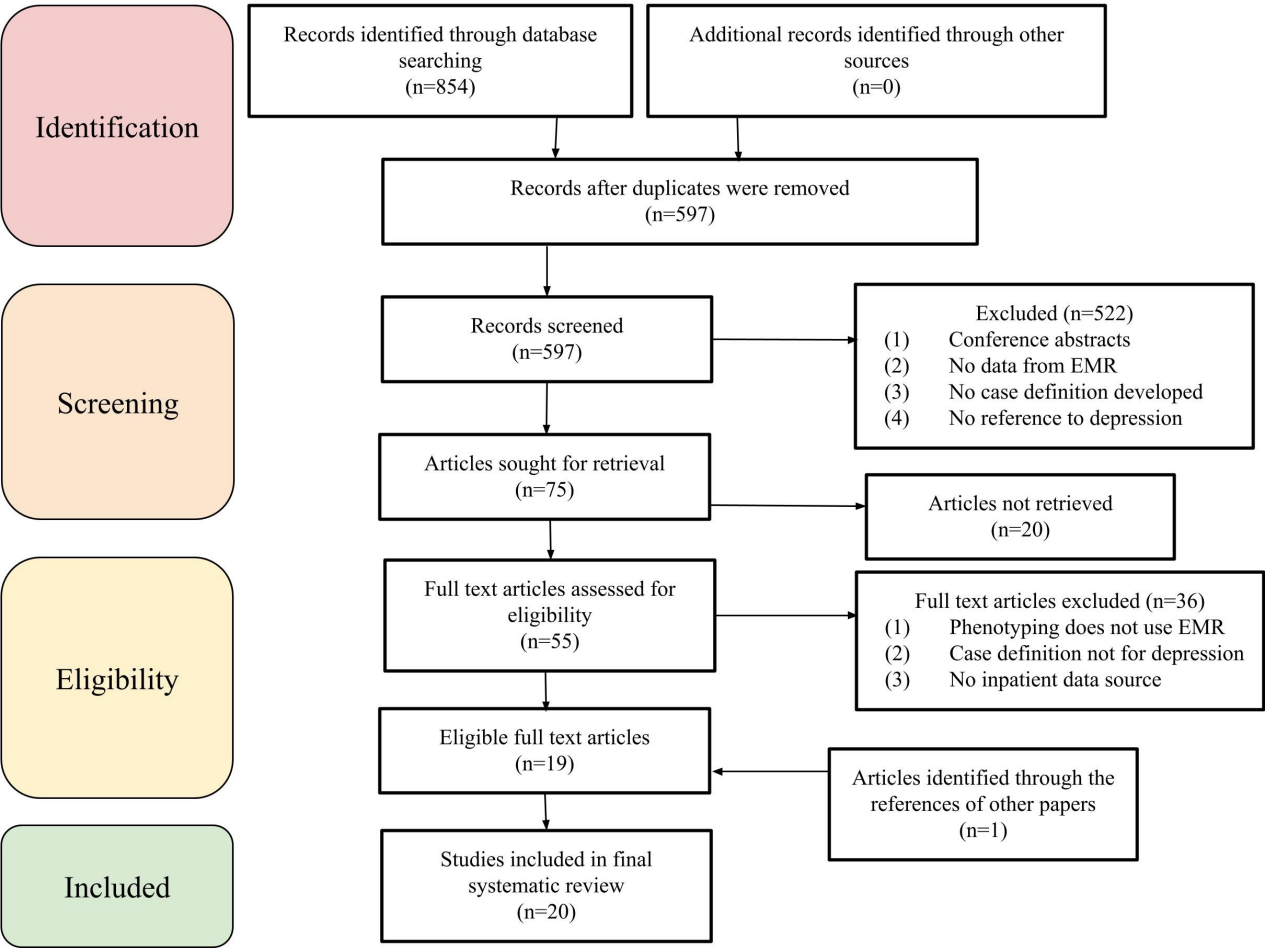
PRISMA flow diagram. EMR: electronic medical record; PRISMA: Preferred Reporting Items for Systematic Reviews and Meta-Analyses.

The database search returned a total of 854 papers. After 257 duplicates were removed, 597 abstracts remained. Then, 522 abstracts were excluded in the title and abstract screening, leaving 75 papers for full-paper review. Of these, 20 papers could not be retrieved, and 36 were excluded based on the exclusion criteria. The 19 remaining papers met all eligibility criteria and were included in the review. Further, 1 additional paper was identified for inclusion from the references of the included papers, resulting in 20 papers for the review [6,19-37]. The PRISMA flow diagram illustrating these steps is shown in Figure 1.

### Characterizing the Identified Literature

Of the 20 studies we identified, the majority occurred in the United States (15/20, 75%). The remaining studies were from the United Kingdom (3/20, 15%) and Spain (2/20, 10%). All the studies were published in 2005 or later.

Most studies looked at inpatient and outpatient data (16/20, 80%), while fewer focused solely on inpatient data (4/20, 20%). A few studies (4/20, 20%) linked EMR data to administrative databases. These studies used structured fields of EMRs and diagnostic codes found in administrative databases. They occurred in 3 countries (United States, United Kingdom, and Spain) and were all published in 2020 or later. Another 3 studies (3/20, 15%) linked EMRs to genomic data (the Partners HealthCare Biobank, United States; the Michigan Genomics Initiative, United States; and the pediatric biorepository database of the Center for Applied Genomics at the Children’s Hospital of Philadelphia, United States). This linkage was conducted in an epidemiological analysis study to find genetic associations between conditions. These characteristics are shown in Table 1.

**Table 1.** Characteristics of included papers.

Paper reference	Country	EMR^a^ setting	Additional data sources used
Dashti et al [19]	United States	Inpatient and outpatient	Genomic data
Dorr et al [20]	United States	Inpatient and outpatient	None
Edgcomb et al [21]	United States	Inpatient and outpatient	None
Estiri et al [22]	United States	Inpatient and outpatient	None
Fang et al [23]	United States	Inpatient and outpatient	Genomic data
Fernandes et al [24]	United Kingdom	Inpatient and outpatient	None
Goulet et al [25]	United States	Inpatient and outpatient	None
Hong et al [26]	United States	Inpatient and outpatient	Administrative data
Ingram et al [27]	United States	Inpatient and outpatient	None
Khapre et al [28]	United Kingdom	Inpatient and outpatient	Administrative data
Mar et al [29]	Spain	Inpatient and outpatient	Administrative data
Mason et al [30]	United Kingdom	Inpatient and outpatient	None
Mayer et al [31]	Spain	Inpatient and outpatient	None
McCoy et al [32]	United States	Inpatient	None
Parthipan et al [33]	United States	Inpatient	None
Perlis et al [6]	United States	Inpatient and outpatient	None
Slaby et al [34]	United States	Inpatient	Genomic data
Tvaryanas et al [35]	United States	Inpatient and outpatient	None
Yusufov et al [36]	United States	Inpatient and outpatient	Administrative data
Zhou et al [37]	United States	Inpatient	None

a EMR: electronic medical record.

Most of the identified studies (18/20, 90%) used diagnostic codes in their case definition for depression. The most common codes used were *ICD-9* (*International Classification of Diseases, Ninth Revision*), followed by *ICD-10* (*International Classification of Diseases, Tenth Revision*). In many studies, the diagnostic code case definitions were combined with structured data elements, such as patient demographics (sex, age, etc), laboratory results, medications, and procedures. For example, procedures were coded with Current Procedural Terminology codes and other types of classifications. Structured EMR data were used in 13/20 studies (65%). Fewer studies (8/20, 40%) incorporated unstructured data elements, such as clinical notes. To analyze these elements, some studies used standardized vocabularies, such as the Unified Medical Language System, to develop lists of keywords. Most studies using unstructured data used NLP techniques to analyze the free-text data in unstructured EMR fields (7/20, 35%). NLP is commonly used on free-text medical data to transform the data into a structured format that can be processed using statistical techniques and machine learning. A quarter of the identified studies (5/20, 25%) used machine learning to develop phenotyping algorithms. Machine learning models included logistic regression, random forest, and propositional rule learners. Table 2 contains details about the algorithms defined in each study.

**Table 2.** Summary of algorithms.

Paper reference	Diagnostic codes?	EMR^a^ – structured?	EMR – unstructured?	ML^b^?	NLP^c^?	Validation methodology	Sensitivity	Specificity	PPV^d^	AUC^e^
Dashti et al [19]	No	Yes	Yes	Yes	Yes	Medical record review	0.81	—^f^	0.90	—
Dorr et al [20]	Yes	Yes	No	No	No	Not specified	—	—	—	—
Edgcomb et al [21]	Yes	No	No	No	No	Not specified	—	—	—	—
Estiri et al [22]	Yes	No	No	No	No	Not specified	—	—	—	—
Fang et al [23]	Yes	Yes	No	No	No	Not specified	—	—	—	—
Fernandes et al [24]	Yes	Yes	No	No	No	Not specified	—	—	—	—
Goulet et al [25]	Yes	No	No	No	No	Medical record review	0.45	0.90	—	—
Hong et al [26]^g^	Yes	Yes	No	Yes	No	Medical record review	—	—	—	0.83
Ingram et al [27]	Yes	Yes	No	No	No	Convergent validity	—	—	—	—
Khapre et al [28]	Yes	Yes	Yes	No	Yes	Not specified	—	—	—	—
Mar et al [29]	Yes	Yes	Yes	Yes	No	Medical record review	—	—	—	0.80
Mason et al [30]	Yes	No	No	No	No	Not specified	—	—	—	—
Mayer et al [31]	Yes	Yes	No	No	No	Not specified	—	—	—	—
McCoy et al [32]	Yes	No	No	No	No	Not specified	—	—	—	—
Parthipan et al [33]	Yes	Yes	Yes	No	Yes	Medical record review	—	—	—	—
Perlis et al [6]	Yes	Yes	Yes	No	No	Medical recordreview	0.39	0.95	0.78	0.87
Slaby et al [34]	Yes	Yes	Yes	No	Yes	Medical recordreview	—	—	0.95	—
Tvaryanas et al [35]	Yes	No	No	No	No	Not specified	—	—	—	—
Yusufov et al [36]	Yes	Yes	Yes	No	Yes	Medical recordreview	0.85	0.95	—	—
Zhou et al [37]	No	No	Yes	Yes	Yes	Medical recordreview	0.87	0.92	—	—

a EMR: electronic medical record.

b ML: machine learning.

c NLP: natural language processing.

d PPV: positive predictive value.

e AUC: area under the receiver operating characteristic curve.

f Not available.

g Area under the precision-recall curve and *F*₁-score were only available for Hong et al [26]. The best algorithm in that paper had an area under the precision-recall curve of 0.90 and an *F*₁-score of 0.81.

Only 9 studies (45%) conducted a medical record review to produce a reference standard to which to compare phenotyping results. Since most of the identified studies (14/20, 70%) were conducted with a larger goal of which phenotyping depression was a small part, many did not provide much information on the methods of their phenotyping. Most studies did not report any metrics measuring the diagnostic accuracy of developed phenotyping algorithms; only 8 studies (40%) reported at least one performance metric. The 6 metrics reported were sensitivity, specificity, positive predictive value (PPV), area under the receiver operating characteristic curve, area under the precision-recall curve, and *F*₁-score. No studies reported negative predictive value. These metrics are displayed in Table 2.

We classified each study into 1 of 3 general purposes: algorithm development, comorbidity analysis, and outcome analysis. A small percentage of the identified studies (6/20, 30%) were conducted for algorithm development. These studies did not look at applications of the phenotyping algorithms developed; instead, they focused on phenotyping methods and algorithm performance. The rest of the studies used a case definition for depression as a step toward a larger goal. For 9 of these studies (9/20, 45%), this goal was outcome analysis or analyzing the effect of depression on patient outcomes, such as mortality, suicide attempts, and psychotherapy receipt. For the remaining studies (5/20, 25%), the goal was comorbidity analysis, examining the prevalence of depression as a comorbidity of other conditions. The comorbidities studied included HIV, hepatitis C, and cancer. Outcome analysis studies have become more prevalent in recent years. Further, 6 were published between 2020 and 2022, up from 3 between 2000 and 2019. In addition, algorithms used for depression phenotyping in EMRs have become more prevalent since 2017.

## Discussion

### Principal Results

In this review, we found 20 papers describing phenotyping algorithms for depression in inpatient EMR data. Most of these algorithms were case definitions using diagnostic codes, specifically *ICD-9*. This reflects that *ICD* (*International Classification of Diseases*) codes are commonly used for billing purposes in the United States and are the most frequently used diagnostic codes in EMRs worldwide [38]. *ICD*-coded data are thus widely available, making them a practical choice when developing a case definition. However, case definitions using diagnostic codes achieved worse sensitivity than algorithms that only used other fields of EMRs. Many algorithms also used structured EMR data [6,19,20,23,24,26-29,31,33,34,36], but fewer used unstructured data [6,19,28,29,33,34,36,37]. NLP and machine learning techniques were used by a minority of algorithms (NLP [19,28,33,34,36,37] and machine learning [19,26,29,37]). These types of machine learning applications are relatively new and are receiving much attention from researchers [39]. The algorithms that used machine learning performed well on all the metrics they reported (sensitivity 0.81‐0.87, specificity 0.82, PPV 0.90, and area under the receiver operating characteristic curve 0.80‐0.83). This suggests that the information in free-text EMR data is valuable for developing accurate phenotyping algorithms. It also supports the effectiveness of machine learning techniques for phenotyping of depression. This is likely an area that will be explored further in future research.

Many of the papers we found did not include a medical record review. If algorithms are not validated against a reference standard such as a medical record review, their accuracy remains unknown. Most papers also did not report metrics measuring the validity of the algorithms developed. This limits the potential of these algorithms for application in precision health care. Conducting validation studies on the algorithms presented in these papers would make them more rigorous. Of the papers that did report metrics, few reported sensitivity, specificity, and PPV together. This could result in skewed interpretations of phenotype performance, as a high sensitivity may come at the cost of a low PPV (or vice versa) for instance.

Based on the validity reported in these papers, an EMR appears promising as a phenotyping tool for depression; however, few studies have reported metrics of diagnostic accuracy of EMR algorithms, especially comprehensive metrics to fully assess performance. Future validation studies conducted on existing case definitions would be valuable in establishing their validity and bringing these types of phenotyping algorithms to the attention of medical professionals and public health analysts. Machine learning and NLP are small but growing areas within phenotyping research. More work could be carried out using these techniques on the unstructured fields in EMRs, alone or in combination with other fields. Finally, as most of the studies we found were performed in the United States on US EMR data, it is to be determined how generalizable the identified case definitions are to data recorded in other jurisdictions. Both the standards of care and the methods of reporting diagnoses vary widely between health care systems, which could result in an algorithm only being valid in the region in which it was developed. There is a need for further research validating existing case definitions across health care regions or creating new case definitions specific to the EMR systems of other countries.

### Limitations

Some relevant papers may have been missed, as we only searched 3 databases. It is also possible that our search terms were not sufficiently broad to return every pertinent paper. We also only considered peer-reviewed papers, not gray literature. However, we developed our search strategy in consultation with librarians and experts in the field with experience performing scoping reviews. For these reasons, we believe our search was sufficient to find papers for the review.

### Conclusions

We examined current algorithms for phenotyping depression in inpatient EMRs. This is an area in which more research needs to be performed. It is difficult to accurately identify cases of depression in EMR data because depression is inconsistently coded, as there is some subjectivity in its diagnosis. Diagnostic codes are primarily used in the algorithms we found, but machine learning on free-text data has recently achieved promising results. Most of the algorithms were developed in the United States; how well they will perform on data from other jurisdictions is yet to be known. In addition, many identified algorithms have yet to be validated against a reference standard, or their performance was not reported. To be useful for public health research, case definitions must be validated; this is an area in which future work is needed. From this study, we conclude that EMRs have the potential to provide valuable insight into the indicators of depression, as well as its prevalence, common comorbidities, and associated outcomes. Future research into applying machine learning and NLP techniques on unstructured EMR data and studies to ascertain the validity and generalizability of existing phenotyping algorithms will be valuable in establishing EMR-based case phenotyping as a reliable tool in precision public health.

## Supplementary material

10.2196/49781 Multimedia Appendix 1 Developed search terms.

10.2196/49781 Multimedia Appendix 2 Summary spreadsheet of identified papers.

10.2196/49781 Checklist 1 PRISMA-ScR (Preferred Reporting Items for Systematic Reviews and Meta-Analyses extension for Scoping Reviews) checklist.

## Abbreviations

EMR: electronic medical record
*ICD*: *International Classification of Diseases*
*ICD-10*: *International Classification of Diseases, Tenth Revision*
*ICD-9*: *International Classification of Diseases, Ninth Revision*
*ICD-9-CM*: *International Classification of Diseases, Ninth Revision, Clinical Modification*
NLP: natural language processing
PPV: positive predictive value
PRISMA-ScR: Preferred Reporting Items for Systematic Reviews and Meta-Analyses extension for Scoping Reviews
