# Supplementary material for: Case Identification of Depression in Inpatient Electronic Medical Records: Scoping Review
Source: JMIR Med Inform. 2024 Oct 14;12:e49781. doi: 10.2196/49781 (PMC11493107; doi:10.2196/49781)
Supplement: Multimedia Appendix 1 [file medinform-v12-e49781-s001.doc]

Search performed May 11, 2023

Databases:

Embase <1974 to 2023 May 10>

Ovid MEDLINE(R) and Epub Ahead of Print, In-Process, In-Data-Review & Other Non-Indexed Citations and Daily <1946 to May 10, 2023>

APA PsycInfo <1806 to May Week 1 2023>

Search Strategy:

| **#** | **Searches** | **Results** |
| --- | --- | --- |
| 1 | electronic medical record* | 119537 |
| 2 | electronic health record* | 100671 |
| 3 | EMR | 32455 |
| 4 | EHR | 30374 |
| 5 | 1 or 2 or 3 or 4 | 225241 |
| 6 | case identification | 3835 |
| 7 | case definition | 12547 |
| 8 | ascertain* | 281681 |
| 9 | phenotyp* | 1961948 |
| 10 | case diagnosis | 2270 |
| 11 | algorithm* | 1151852 |
| 12 | 6 or 7 or 8 or 9 or 10 or 11 | 3363948 |
| 13 | depress* | 2033848 |
| 14 | dysthymi* | 18711 |
| 15 | adjustment disorder* | 16030 |
| 16 | 13 or 14 or 15 | 2044080 |
| 17 | 5 and 12 and 16 | 854 |
